# ANTERIOR SEGMENT OPTICAL COHERENCE TOMOGRAPHY IN DETERMINATION OF ENTRY SITE FOR VITRECTOMY IN HIGHLY MYOPIC EYES

**DOI:** 10.1097/IAE.0000000000003736

**Published:** 2023-02-16

**Authors:** Kazushi Hirono, Maiko Inoue, Shin Tanaka, Eiichi Uchio, Yasuo Yanagi, Kazuaki Kadonosono

**Affiliations:** * Department of Ophthalmology and Micro-technology, Yokohama City University; and; † Department of Ophthalmology, Fukuoka University.

**Keywords:** anterior segment OCT, axial length, ciliary body, myopic traction maculopathy, myopia, pars plana, sclerotomy

## Abstract

To evaluate the efficacy of anterior segment optical coherent tomography (AS OCT) in estimating the length of the pars plana and optimizing the sclerotomy entry site in vitrectomy for highly myopic eyes, facilitating membrane peeling.

**Methods:**

Twenty-three eyes with myopic traction maculopathy were studied. The pars plana was examined using two methods: preoperative AS OCT and intraoperative measurement. The distance from the limbus to the ora serrata in two groups was measured to compare differences in length. The actual length of the entry site from the limbus and forceps used were noted in all eyes studied.

**Results:**

The mean axial length was 29.2 ± 2.3 mm for all 23 eyes. The average length between the limbus and ora serrata measured with AS OCT and intraoperative examination was 6,710 *µ*m (SD ± 459) and 6,671 *µ*m (SD ± 402), respectively, in the superotemporal region (*P* > 0.05), and 6,340 *µ*m (SD ± 321) and 6,204 *µ*m (SD ± 402), respectively, in the superonasal region (*P* >0.05). The mean length of the entry site from the limbus was 6.2 mm, and 28-mm forceps were used in 17 of 23 eyes (77%).

**Conclusion:**

The length of the pars plana varies depending on the axial length of the eye. Preoperative AS OCT enables accurate measurement of the pars plana in eyes with high myopia. AS OCT examination can help determine the optimal site for sclerotomy, allowing easier access to the macular region for membrane peeling in highly myopic eyes.

## Abstract 2

Preoperative anterior segment optical coherent tomography enables accurate measurement of the pars plana in eyes with high myopia. Anterior segment optical coherent tomography examination can help determine the optimal site for sclerotomy, allowing easier access to the macular region for effective membrane peeling in highly myopic eyes.

Since the advent of vitrectomy, the entry sites where surgical instruments are inserted through sclerotomy have been placed at the pars plana.^1^ It is widely accepted that the entry site should be created in the region of the pars plana 4.0 mm away from the limbus and at a shorter distance in pseudophakic or aphakic eyes and pediatric patients.^2^ The pars plana, the posterior portion of the ciliary body, is contiguous to the choroid at the ora serrata where there are few surgical complications related with sclerotomy in vitrectomy.^3^ The length of the pars plana when measured in vivo with anterior segment optical coherence tomography (OCT), varies significantly among eyes with different axial lengths.^4^

Recently, there are an increasing number of patients with highly myopic eyes^5,6^ presenting with conditions such as myopic traction maculopathy or retinal detachment.^7–9^ However, it is very difficult to perform membrane peeling in those eyes due to the increased axial length.^10–12^ There are a number of techniques possible to resolve this problem, including removal of the cannula, indentation of the eye, and using forceps with a longer shaft. However, the method of placing the entry site closer to the macular region provides better access to the retina.^13^ Theoretically, it is possible to have the entry site at a point much further than 4.0 mm from the limbus in highly myopic eyes because the ciliary body is longer in these patients. Nevertheless, it seems surgeons are reluctant to place the entry site of vitrectomy further than 4.0 mm without any preoperative estimation, mainly because of iatrogenic retina breaks and subsequent retinal detachment.^14,15^

Anterior segment OCT (AS OCT) is an innovative tool for evaluating eye structure in anterior segments.^16^ As improvements in the technology have been made, such as the speed and resolution of the images captured, the impact of AS OCT imaging as a tool in clinical practice has been rising. In addition, advances in OCT image-processing software^17^ allow for multiple scans, three-dimensional reconstruction, and more accurate measurements. Although AS OCT has been used for anterior eye structures such as the cornea, the anterior chamber, and the angle, there have been few reports regarding the application of AS OCT to pars plana measurement. If anterior OCT allows accurate estimates of the pars plana region preoperatively in highly myopic eyes, we could minimize possible risks related with relocation of the entry site and perform membrane peeling more easily.

In this study, we conducted research to evaluate the accuracy of preoperative anterior OCT for measurement of the pars plana and compared data with intraoperative observation of the pars plana, with the goal of facilitating membrane peeling during vitrectomy, while minimizing risks associated with entry sites placed further than 4.0 mm from the limbus.

## Methods

This study was conducted at Yokohama City University Medical Center, and all procedures used in this study were approved by the ethics committee of the university and adhered to the tenets of the Declaration of Helsinki. Twenty-three eyes with myopic traction maculopathy were included in this prospective study between January 2022 and June 2022. From each individual, written informed consent was obtained. Male and female patients older than 18 years were included. Eyes were excluded based on prior intravitreal injections, history of vitrectomy, pathologies of the sclera, or macular pathologies. Pseudophakic eyes (n = 8) were not excluded because they did not have prior surgery affecting the sclera. A spectral domain AS OCT (CASIA2, Tomey Corp, Tokyo, Japan) was used to image the ciliary body and the anterior sclera including the limbus. Ambient illumination was reduced to a minimum to avoid interfering signals. To obtain images of the anterior segment in one quadrant, all participants were asked to look at a fixation light located to the superotemporal image. A broad segment of the anterior structures of the eye, a scanned area with a pattern size of 15° × 5° (8.3 × 2.8 mm) was partially placed over the limbus. Each scanned area included 11 B-scans, which were separated by 277 *µ*m. Automatic real-time function was set at 36 frames to achieve superior resolution.

**Fig. 1.**
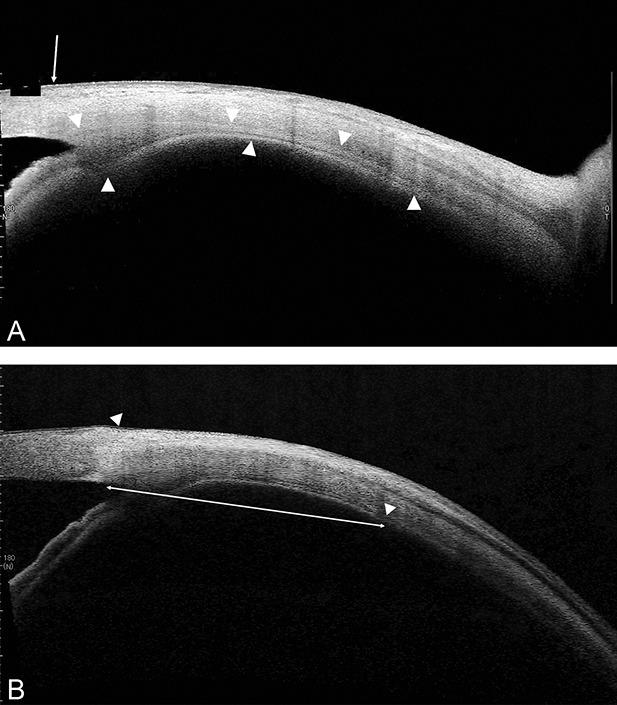
An image of AS OCT for the pars plana. **A.** Ciliary body (arrowheads) and the limbus (arrow) are seen. **B.** An arrow shows the line from the limbus (left arrowhead) to the end of ciliary body (right arrowhead). The length is 7,020 *µ*m.

The length between the limbus and the end of ciliary body (which is equivalent to the ora serrata) was measured using the CASIA software (Figure 1). Measurements were performed by two independent examiners in three different B-scans of the superotemporal and superonasal regions, after which the measurements were averaged. The mean calculated intraclass correlation coefficient between the individual measurements was 0.91 (95% confidence interval, 0.89–0.93).

The ciliary body length was measured as the distance in a straight line from the deepest point of the iridotrabecular angle to the last discernible mass of the ciliary body. The mass was defined as the region where there was no gap between the ciliary epithelium and the sclera, and after this point, the ciliary body epithelium or internal limiting membrane of retina continued parallel to the sclera.

**Fig. 2.**
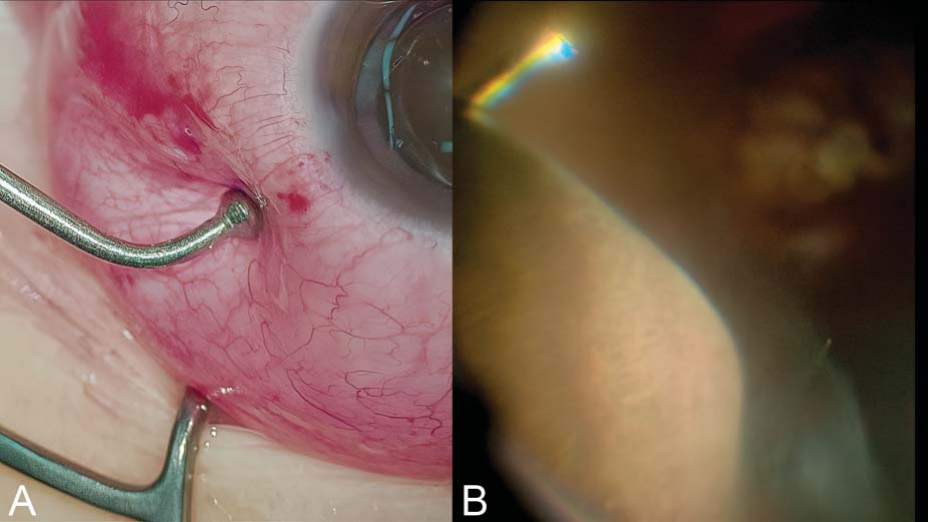
Intraoperative measurement of the pars plana. The ora serrata is indented with the Meyer-Schwickerath instrument (**A**), the orientation of which was correctly confirmed intraoperatively during vitrectomy (**B**). The length from the limbus to the ora serrata was 7,300 *µ*m. These data were compared with that from AS OCT.

Each eye was examined intraoperatively during vitrectomy to measure the length between the limbus and ora serrata (Figure 2). The ora serrata in the superior temporal and nasal regions was directly marked with a scleral indentation by one surgeon using a Meyer-Schwickerath instrument, and then, the length of the sclera in this region was calculated with a caliper, in conjunction with a micrometer (Shinwa Measuring Tools Corp. Japan). These measurements were performed twice to get an average length.

The actual length between the limbus and sclerotomy, and length of forceps used were noted in all eyes studied.

The lengths of the pars plana obtained from both anterior OCT and intraoperative examination were analyzed statistically to compare the results. Data were analyzed for normality using the Pearson test. For comparison of groups, analysis of variance (ANOVA) was used after verifying normality. A *P* value of 0.05 or smaller was considered statistically significant.

## Results

Of the 23 eyes enrolled in this study, there were 14 women and nine men The mean age was 63.2 years (SD ± 11.4, range 45–72 years). The mean axial length was 29.24 mm (SD ± 2.32, range 27.68–32.90 mm) (Table 1).

**Table 1.** Patient Data

		Age/Sex/Eye	Axial length (mm)	AS OCT (mm)		Intraoperative (mm)		Entry site (mm)	Forceps used (mm)
				T	N	T	N		
1.	RE	56/F/RE	28.92	6.72	6.27	6.34	5.90	6.0	28
2.	RE	62/M/RE	28.12	6.34	6.00	6.02	5.50	6.0	28
3.	LE	56/F/LE	30.12	7.12	6.78	6.89	6.56	6.5	28
4.	LE	67/F/LE	31.12	7.20	7.00	7.01	6.40	6.5	28
5.	RE	57/M/RE	27.89	6.78	6.09	6.45	6.12	6.5	28
6.	RE	65/F/RE	28.03	6.32	5.55	7.01	6.23	6.0	28
7.	RE	64/M/RE	29.09	7.19	7.00	7.24	6.98	6.5	32
8.	LE	71/F/LE	30.03	6.90	6.80	7.31	6.78	6.5	32
9.	RE	56/F/RE	29.99	6.78	6.08	6.90	6.50	6.0	28
10.	LE	67/M/LE	30.12	7.23	7.01	7.45	6.78	7.0	28
11.	RE	74/M/RE	28.89	5.40	5.40	5.98	5.67	5.0	28
12.	LE	62/F/LE	29.67	6.99	6.34	6.78	6.22	6.5	28
13.	RE	72/F/RE	27.68	5.89	5.44	6.78	6.45	5.0	32
14.	LE	70/M/LE	28.07	6.54	6.23	6.02	5.56	6.0	28
15.	RE	54/F/RE	27.23	6.90	6.78	5.67	5.21	6.0	28
16.	RE	58/F/RE	30.19	7.24	7.12	7.02	6.34	6.0	32
17.	RE	69/M/RE	28.79	6.78	6.18	6.99	6.89	6.5	28
18.	RE	70/F/RE	29.90	7.02	6.56	6.09	5.56	6.0	32
19.	RE	65/M/RE	27.90	5.98	5.45	6.02	5.89	5.5	28
20.	RE	69/F/RE	29.09	6.50	6.23	6.34	6.12	6.5	28
21.	RE	72/F/RE	31.01	7.34	7.12	7.56	6.35	7.0	28
22.	LE	48/F/LE	27.80	5.89	5.00	6.54	6.34	5.5	28
23.	RE	45/F/RE	32.90	7.32	7.30	7.02	6.30	7.0	28
									
		63.0	29.24	6.712	6.336	6.671	6.202	6.2	

AS OCT, the length from the limbus to pars plana by anterior segment OCT. Intraoperative, the length from the limbus and pars plana measured intraoperatively. Entry site, the actual length from the limbus to sclerotomy. Forceps used, the length of forceps used intraoperatively.

RE, right eye. LE, left eye. T, temporal. N, nasal.

The mean temporal and nasal length from the limbus to the end of ciliary body using anterior OCT was 6,710 *µ*m (SD ±459) and 5,610 *µ*m (SD ± 332), respectively, while the mean temporal and nasal length from the limbus to the ora serrata according to intraoperative examination was 6,671 *µ*m (SD ± 402) and 5,700 *µ*m (SD ± 390), respectively. There was no significant difference between the two groups in either temporal or nasal length (*P* = 0.151, 0.221) (Table1).

**Fig. 3.**
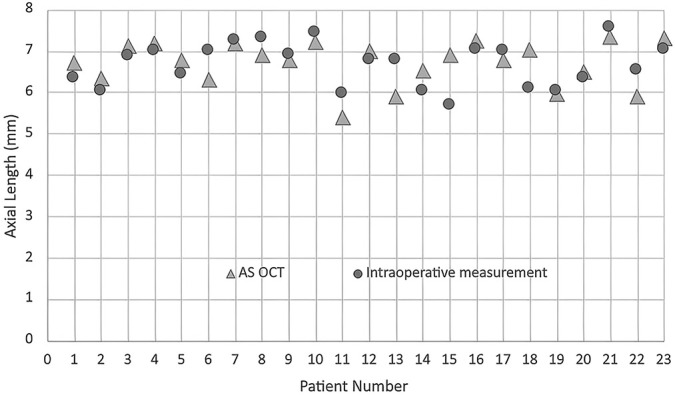
A graph of the length from the limbus to the ora serrata in 23 eyes studied in anterior segment OCT and intraoperative measurement in the temporal region. The difference in the length from the limbus to the ora serrata between AS OCT and intraoperative actual length was within 500 *µ*m in all eyes studied in the temporal region.

**Fig. 4.**
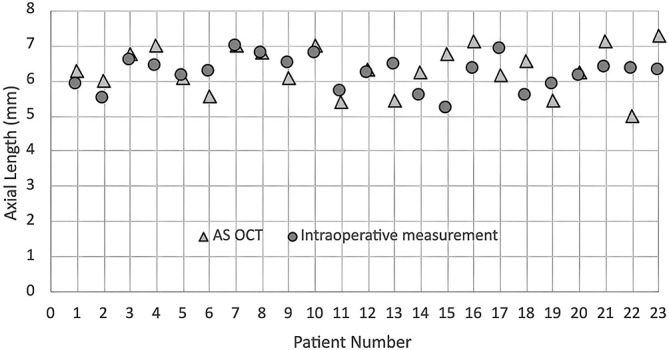
A graph of the length from the limbus to the ora serrata in 23 eyes studied in anterior segment OCT and intraoperative measurement in the nasal region. The difference in the length from the limbus to the ora serrata between AS OCT and intraoperative actual length was within 500 *µ*m in all eyes studied in nasal regions.

The average difference in the length from the limbus to the ora serrata between preoperative anterior OCT and intraoperative measurement on temporal and nasal region was 140 *µ*m (SD ± 45) and 204 *µ*m (SD ± 31), respectively (Figures 3 and 4).

The mean length of the entry site from the limbus in all eyes studied was 6.2 mm, and 28-mm forceps (MaxGrip Alcon Laboratories) were used during membrane peeling in 17 eyes (77%) (Table 1). There were not any severe intraoperative surgical complications such as an iatrogenic break related to the increased distance of the sclerotomy site from the limbus.

## Case

**Fig. 5.**
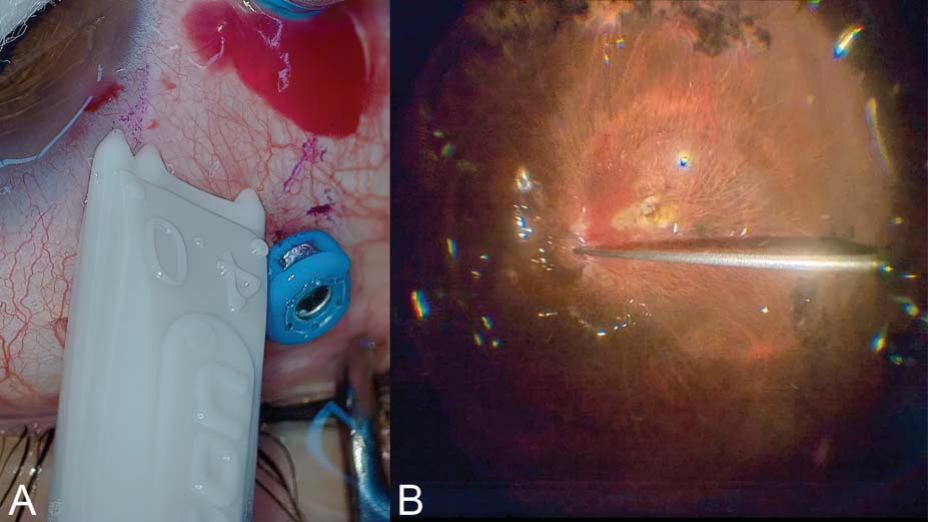
The entry site and intraoperative view. Sclerotomy was marked at 6.5 mm from the limbus (**A**). A 28-mm long soft-tip cannula was inserted from the posterior end of the pars plana during fluid–air exchange to reach the optic disk (**B**).

A 56-year-old woman with retinoschisis due to myopic traction maculopathy underwent vitrectomy (No 3, Table 1). Her visual acuity was 20/400, and the axial length was 30.12 mm in the right eye. Preoperative examination using anterior OCT showed that the length from the limbus to the end of ciliary body was 7120 mm, while intraoperative examination of the length from the limbus to the ora serrata measured 6890 mm. After a sclerotomy was created at 6.5 mm from the limbus, membrane peeling was effectively performed with 28-mm forceps (Figure 5). Postoperative examination revealed that foveal thickness was reduced, and visual acuity improved to 20/200 without any surgical complications such as retinal detachment.

## Conclusion

We measured the length from the limbus to the end of the ciliary body using preoperative anterior OCT and compared this with the length from the limbus to the ora serrata measured intraoperatively. The results showed that lengths measured using both methods were statistically the same, suggesting that anterior OCT examination is effective for preoperatively estimating the length of the pars plana in highly myopic eyes. Because the length of the pars plana varies with axial length,^18^ it is important to know the length of the pars plana before surgery to safely place sclerotomy during vitrectomy in highly myopic eyes.^14,15^

It is a challenging but essential procedure to peel fragile membranes such as the epiretinal membrane and internal limiting membrane in highly myopic eyes.^12^ It is common for the eye to be too long for surgeons to reach the retinal surface with forceps of normal length in patients with high myopia, especially when the axial length is more than 30 mm,^19^ and it becomes difficult to perform delicate surgical procedures due to the flexibility of forceps with a longer shaft. Some surgeons place the trochar at a site further from the limbus such as at 5 or 6 mm^13^ to facilitate access to the retina in highly myopic eyes. However, there may be an increased risk of retinal damage when performing these kinds of procedures without any clear observation during surgery.

We find preoperative examination of the pars plana using anterior OCT to be useful in patients with highly myopic eyes requiring pars plana vitrectomy. It provides us with an accurate estimate of pars plana length enabling safe placement of the sclerotomy while also allowing the most posterior entry into the vitreous chamber providing easier access to the retina in these myopic eyes.

There are limitations to this study. We used the latest anterior OCT machine, allowing us to obtain high-resolution images. It requires a learning curve to correctly measure the ciliary body based on the results of anterior OCT; however, it might be possible to apply algorithms or AI to these measurements in the future. In addition, the number of eyes studied was relatively low for a statistical examination.

In conclusion, we investigated the efficacy of AS OCT in measuring the pars plana to optimize the entry site in vitrectomy for myopic eyes. AS OCT enables accurate preoperative measurement of the pars plana in highly myopic eyes. Preoperative AS OCT examination can help determine the optimal site of sclerotomy, allowing surgeons easier access to the macular region for effective membrane peeling in highly myopic eyes.

## References

[1] Buettner H Machemer R. Histopathologic findings in human eyes after pars plana vitrectomy and lensectomy. Arch Ophthalmol 1977;95:2029–2033. 921582 10.1001/archopht.1977.04450110123015

[2] Machemer R Norton EW. Vitrectomy, a pars plana approach. II. Clinical experience. Mod Probl Ophthalmol 1972;10:178–185. 5056324

[3] Delamere NA. Ciliary body and ciliary epithelium. Adv Organ Biol 2005;10:127–148. 21234280 10.1016/S1569-2590(05)10005-6 PMC3018825

[4] Lincke JB Keller S Amaral J . GraefesCiliary body length revisited by anterior segment optical coherence tomography: implications for safe access to the pars plana for intravitreal injections. Arch Clin Exp Ophthalmol 2021;259:1435–1441. 10.1007/s00417-020-04967-3 PMC8166736 33074373

[5] Holden BA Fricke TR Wilson DA . Global prevalence of myopia and high myopia and temporal trends from 2000 through 2050. Ophthalmology 2016;123:1036–1042. 26875007 10.1016/j.ophtha.2016.01.006

[6] Ohno-Matsui Kyoko Wu Pei-Chang Yamashiro Kenji . IMI pathologic myopia. Invest Ophthalmol Vis Sci 2021;62:5 10.1167/iovs.62.5.5 PMC8083114 33909033

[7] Phillips CI. Retinal detachment at the posterior pole. Br J Ophthalmol 1958;42:749–753. 13607958 10.1136/bjo.42.12.749 PMC509747

[8] Takano M Kishi S. Foveal retinoschisis and retinal detachment in severely myopic eyes with posterior staphyloma. Am J Ophthalmol 1999;128:472–476. 10577588 10.1016/s0002-9394(99)00186-5

[9] Panozzo G Mercanti A. Vitrectomy for myopic traction maculopathy. Arch Ophthalmol 2007;125:767–772. 17562987 10.1001/archopht.125.6.767

[10] Russell JF Naranjo A Dubovy SR Smiddy WE. Clinicopathologic correlation of preretinal tissues in myopic traction maculopathy. Retina 2021;41(7):1512–1517. 33239545 10.1097/IAE.0000000000003045

[11] Johnson MW. Myopic traction maculopathy: pathogenic mechanisms and surgical treatment. Retina 2012;32:S205–S210. 22929322 10.1097/IAE.0b013e31825bc0de

[12] Parolini B Palmieri M Finzi A Frisina R. Proposal for the management of myopic traction maculopathy based on the new MTM staging system. Eur J Ophthalmol 2021;31:3265–3276. 33345597 10.1177/1120672120980943

[13] Iwama Y Ikeda T Nakashima H . Extending the limbus-to-cannula distance to 6.0 mm during pars plana vitrectomy in highly myopic eyes. Retina 2022;42:1199–1202. 34077167 10.1097/IAE.0000000000003025

[14] Scartozzi R Bessa AS Gupta OP Regillo CD. Intraoperative sclerotomy-related retinal breaks for macular surgery, 20- vs 25-gauge vitrectomy systems. Am J Ophthalmol 2007;143:155–156. 17188054 10.1016/j.ajo.2006.07.038

[15] Böker T Spitznas M. Ultrasound biomicroscopy for examination of the sclerotomy site after pars plana vitrectomy. Am J Ophthalmol 1994;118:813–815. 7977614 10.1016/s0002-9394(14)72567-x

[16] Ang M Baskaran M Werkmeister RM Anterior segment optical coherence tomography. Prog Retin Eye Res 2018;66:132–156. 29635068 10.1016/j.preteyeres.2018.04.002

[17] Wang J Abou Shousha M Perez VL . Ultra-high resolution optical coherence tomography for imaging the anterior segment of the eye. Ophthalmic Surg Lasers Imaging 2011;42:S15–S27. 21790108 10.3928/15428877-20110627-02

[18] Lemley CA Han DP. An age-based method for planning sclerotomy placement during pediatric vitrectomy: a 12-year experience. Trans Am Ophthalmol Soc 2007;105:86–89. 18427597 PMC2258105

[19] Gao X Ikuno Y Nishida K. Long-shaft forceps for membrane peeling in highly myopic eyes. Retina 2013;33:1475–1476. 23751945 10.1097/IAE.0b013e318297f85b

